# LABS: linear amplification-based bisulfite sequencing for ultrasensitive cancer detection from cell-free DNA

**DOI:** 10.1186/s13059-024-03262-2

**Published:** 2024-06-14

**Authors:** Xiao-Long Cui, Ji Nie, Houxiang Zhu, Krissana Kowitwanich, Alana V. Beadell, Diana C. West-Szymanski, Zhou Zhang, Urszula Dougherty, Akushika Kwesi, Zifeng Deng, Yan Li, Danqing Meng, Kevin Roggin, Teresa Barry, Ryan Owyang, Ben Fefferman, Chang Zeng, Lu Gao, Carolyn W. T. Zhao, Yuri Malina, Jiangbo Wei, Melanie Weigert, Wenjun Kang, Ajay Goel, Brian C.-H. Chiu, Marc Bissonnette, Wei Zhang, Mengjie Chen, Chuan He

**Affiliations:** 1 Department of Chemistry, Department of Biochemistry and Molecular Biology, Institute for Biophysical Dynamics, The University of Chicago, Chicago, IL USA; 2 Howard Hughes Medical Institute, The University of Chicago, Chicago, IL USA; 3 Department of Preventive Medicine, Northwestern University Feinberg School of Medicine, Chicago, IL USA; 4 Department of Medicine, The University of Chicago, Chicago, IL USA; 5 Department of Human Genetics, The University of Chicago, Chicago, IL USA; 6 Department of Molecular Biosciences, Northwestern University, Evanston, IL USA; 7 Department of Surgery, The University of Chicago, Chicago, IL USA; 8 Department of Obstetrics and Gynecology/Section of Gynecologic Oncology, The University of Chicago, Chicago, IL USA; 9 City of Hope Comprehensive Cancer Center, Duarte, CA USA; 10 Department of Public Health Sciences, The University of Chicago, Chicago, IL USA; 11 The Robert H. Lurie Comprehensive Cancer Center, Northwestern University Feinberg School of Medicine, Chicago, IL USA

**Keywords:** Linear amplification, Bisulfite sequencing, Low-input DNA, Cell-free DNA, Cancer detection

## Abstract

Methylation-based liquid biopsies show promises in detecting cancer using circulating cell-free DNA; however, current limitations impede clinical application. Most assays necessitate substantial DNA inputs, posing challenges. Additionally, underrepresented tumor DNA fragments may go undetected during exponential amplification steps of traditional sequencing methods. Here, we report linear amplification-based bisulfite sequencing (LABS), enabling linear amplification of bisulfite-treated DNA fragments in a genome-wide, unbiased fashion, detecting cancer abnormalities with sub-nanogram inputs. Applying LABS to 100 patient samples revealed cancer-specific patterns, copy number alterations, and enhanced cancer detection accuracy by identifying tissue-of-origin and immune cell composition.

**Supplementary Information:**

The online version contains supplementary material available at 10.1186/s13059-024-03262-2.

## Background

Early tumor detection is one of the major factors in successful cancer treatment that could significantly reduce cancer-related mortality and healthcare cost burden. Liquid biopsies are gaining prominence as a method for early cancer detection and management because they are minimally invasive compared to tissue biopsies. Most liquid biopsy-based methods employ circulating cell-free DNA (cfDNA) from plasma, which is composed of circulating tumor DNA (ctDNA) as well as background non-ctDNA [1–3]. Reflected in ctDNA fragments, released from the tumor into the blood, are a range of molecular alterations occurring in tumor cells, such as somatic point mutations, copy number alterations, and cytosine modifications. Non-ctDNA also contains critical information such as tumor microenvironment and patient immune cell composition.

The first generation of sequencing-assisted liquid biopsy assays relied on identifying specific somatic mutations including actionable mutations from cfDNA [4, 5]. However, the diagnostic scope of these assays has been limited for tumors with low mutation rates like hematological cancers and tumor types that lack recurrent mutations due to their inherent heterogeneity. As low mutation rates have limited cancer detection using liquid biopsies, researchers have been focusing on the analysis of DNA methylation (5-methylcytosine or 5mC), a crucial epigenetic modification relevant to cancer development. In the past decade, several DNA methylation-based assays have been proposed for early cancer detection using cfDNA. Comprehensive 5mC profiles hold the promise of simultaneously detecting cancer and identifying the tissue of origin (TOO) through genome-wide analysis of alterations in methylation patterns [3, 6–13]. Whole-genome bisulfite sequencing (WGBS) is the “gold standard” for measuring DNA methylation at a single base pair (bp) resolution. Cost-effective targeted bisulfite sequencing (TBS) was also developed to direct sequencing towards more informative parts of the genome through target-specific enrichment of regions using probe hybridization capture or non-specific enrichment of CpG-dense regions by reduced-representation bisulfite sequencing (RRBS). Unfortunately, all these existing methods require large amounts of input DNA (Illumina TruSeq requiring at least 250 ng, NuGen RRBS requiring at least 100 ng) that are often prohibitive for clinical biospecimens, thus hampering methylome-based assays from being used in “real-world” clinical applications. For example, the existing methods cannot be used in clinically feasible volumes of plasma (e.g., a few milliliters) that contain probably less than 10 ng of cfDNA as the input. Additionally, ctDNA from tumors or cfDNA reflecting tumor-adjacent tissues may be present at even lower levels in the total cfDNA. These tumor-related components of cfDNA are often underrepresented in the current DNA methylation assays because exponential amplification is used in sample treatments, leading to reduced detection coverage and sensitivity.

To address the challenges posed by complex and DNA-limited clinical samples, we have developed a new assay called linear amplification-based bisulfite sequencing (LABS). This new procedure can linearly amplify 5mC signals, making it possible to analyze sub-nanogram amounts of input DNA. Our benchmarking experiments demonstrated that the LABS could robustly profile genome-wide 5mC at single base-pair resolution, with an input amount as low as 10 pg, thereby overcoming the limitations of conventional assays. For cfDNA samples, the LABS preserves underrepresented DNA components and copy number aberrations, which could add an extra dimension of signals for cancer detection. The linear amplification further enables TOO identification and immune cell deconvolution, therefore improving the power of methylation-based cfDNA analysis. Using plasma-derived cfDNA samples from a set of 100 cfDNA samples, including 50 from colorectal cancer (CRC) donors, 16 from pancreatic ductal adenocarcinoma (PDAC) donors, and 34 from healthy controls, we demonstrated that the LABS enabled accurate detection of patients with different cancer types, by integrating methylation, copy number, and cellular deconvolution results from the LABS data.

## Results

### Scheme of the LABS

**Fig. 1:**
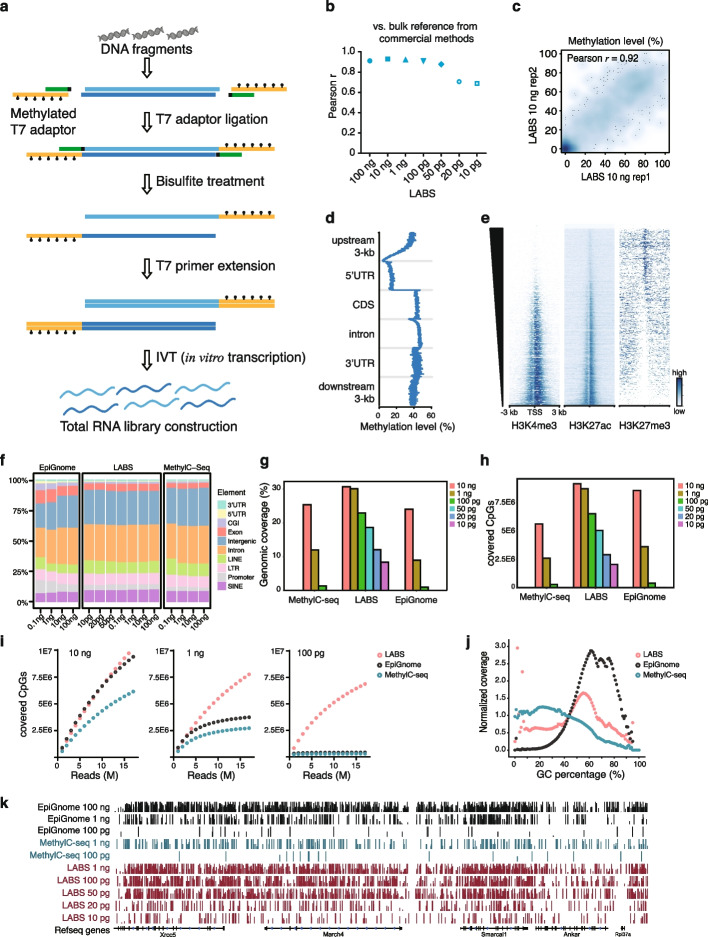
Development and validation of the LABS. **a** The schematic for the LABS. Fully methylated T7 adaptors were ligated to each end of DNA fragments and then treated with bisulfite (BS). The promoter region was further annealed and extended with a complementary T7 primer and then initiated in vitro transcription (IVT). **b** High correlation between the LABS and bulk reference. **c** High correlation between the LABS replicates. **d** Metagene plot of the LABS profiles showing low methylation levels at promoters and high methylation levels at gene bodies. **e** Heatmaps showing negative correlations of the LABS profiles with H3K4me3 and H3K27ac signals and positive correlations with H3K27me3 signals at TSS flanking regions. TSS, transcription start sites. **f** Annotation of covered CpGs for all three methods from different input DNA amounts. **g** Genomic coverage of the LABS from different amounts of input DNA, compared to the two competing methods. **h** Covered CpGs of the LABS from different amounts of input DNA, compared to the two competing methods. **i** Saturation curves of the LABS from different amounts of input DNA, compared to the two competing methods. **j** Distribution curves showing that the LABS has better coverage at regions with extreme GC percentages. **k** Genome browser view showing the LABS profiles from different amounts of input DNA, compared with the two competing methods

In the LABS protocol, a fully methylated T7 adaptor is ligated to each end of the DNA fragment, and the resulting construct is treated with bisulfite. The T7 adaptor includes a T7 RNA polymerase promoter sequence and a 3′-end blocked short helper to form a partial double-stranded DNA structure for ligation. During the bisulfite treatment, all cytosines (C) are converted into uracils (U), while the 5mC remains unchanged. The T7 promoter sequence remains intact as well. After the bisulfite treatment, the promoter region is annealed and extended with a complementary T7 primer, which will initiate in vitro transcription (IVT). In IVT amplification, trace amounts of DNA fragments will be evenly amplified into multiple RNA copies. The RNA products will be subjected to reverse transcription, second-strand synthesis, adaptor ligation, library amplification, and sequencing (Fig. 1a).

The LABS protocol avoids potential amplification bias that could be introduced by conventional protocols of strand displacement. The single-stranded DNAs (ssDNAs) are generated linearly from 5′- to 3′-ends or to the bisulfite-damaged nicking bases, by tagging the T7 adaptor before bisulfite treatment and initializing T7 RNA polymerase-based IVT after bisulfite treatment. Therefore, the LABS approach helps preserve genomic information and measures the entire methylome in an unbiased fashion.

### Technical robustness of the LABS

To test how well the LABS performed with varying amounts of input materials and identify any technical limitations, we used 100 ng, 10 ng, 1 ng, 100 pg, 50 pg, 20 pg, and 10 pg of genomic DNA (gDNA) from the E14Tg2a mouse embryonic stem cells (mESCs) for the experiment, with 2 biological replicates for each amount (Additional file 1: Fig. S1a, Additional file 2: Table S1). Each library generated an average of 31.3 million reads (with a standard deviation of 6.2 million). We found consistent CpG methylation levels across all samples, averaging at 42.7%, while CHG and CHH (where H is A, C, or T) methylation levels were low, averaging at 0.81% and 0.72%, respectively (Additional file 1: Fig. S1b). The comparison revealed a high level of accuracy in the LABS results, with a Pearson correlation coefficient of 0.88 for 10 ng input DNA (Fig. 1b). We also found that the biological replicates of the LABS data showed high reproducibility, with a Pearson correlation coefficient of 0.92 (Fig. 1c). Relatively low DNA methylation levels were observed at promoter regions and relatively high levels at gene body regions, which were reported to repress initiation of spurious transcription and ensure transcription fidelity, consistent with known DNA methylation patterns (Fig. 1d) [14–16]. Integration of the LABS profiles with histone modification profiles in mESCs revealed negative correlations between DNA methylation and H3K4me3/H3K27ac and a positive correlation between DNA methylation and H3K27me3, indicating high specificity of the LABS data (Fig. 1e, Additional file 1: Fig. S1d). Further annotations of the LABS-covered CpGs indicated that this method reliably captured genome-wide CpGs, with the majority of them found to be located in intronic (30.0% on average) and intergenic regions (27.7% on average) (Fig. 1f).

To further assess the performance of the LABS compared to the existing methods, we performed whole-genome bisulfite sequencing (WGBS) on the same mESC line using two commercially available methods, i.e., MethylC-seq and EpiGnome, with input amounts of 100 ng, 10 ng, 1 ng, and 100 pg of mESC gDNA (Additional file 1: Fig. S1a). The libraries were sequenced to comparable sizes, averaging 44.9 ± 10.3 million reads per library.

It is noted that high duplication rates are commonly observed for WGBS libraries from low-input DNA, thus resulting in limited useful information and hampering the application of these assays to low-input cfDNA samples. We compared the duplication rates using different methods. We found that LABS displayed the lowest duplication levels across all input DNA amounts (Additional file 1: Fig. S1g). In contrast, MethylC-seq and EpiGnome showed extremely high duplication rates when using 100 pg input DNA, with rates of 96.87% and 97.47%, respectively. In comparison, the LABS achieved a duplication ratio as low as 9.72% for the same amount of input DNA. Furthermore, despite limiting at 10 pg of input DNA, only 40.77% of reads were duplicates for the LABS, indicating a dramatically improved detection limit compared to existing methods.

Next, we evaluated the coverage across the different methods, as high coverage rate is critical to obtain sufficient information, especially for low abundant DNA components. Overall, the LABS showed higher coverage of CpGs, genomic regions, and chromosomes, compared to the existing methods (Fig. 1g, h, and Additional file 1: Fig. S1e) at the same amount of input DNA. The coverage decreased slightly with reduced input DNA, with an average decrease of 12.9% for a tenfold reduction in input from 10 ng to 100 pg. In contrast, the coverage decreased by 70.9% for EpiGnome and 75.8% for MethylC-seq under the same conditions.

Coverage bias towards GC-rich regions has been reported as a drawback for the WGBS methods, which can be attributed to incomplete bisulfite conversion, PCR amplification bias, and some polymerases used in library construction [17]. Of the three methods evaluated in this study, the LABS showed the highest uniformity across regions with varying percentages of GC content and maintained good performance even with only 100 pg of input DNA (Fig. 1j). The LABS also significantly reduced bias towards GC content compared to the two competing methods, achieving high coverage for extreme regions (GC percentages > 80% or < 20%). The coverage patterns on both DNA strands were comparable for all three methods (Additional file 1: Fig. S1c).

In line with the low duplication levels, high genomic coverage, and low coverage bias observed in this study, the LABS demonstrated slow saturation with increasing input DNA amounts, resulting in greater library complexities (Fig. 1i, k, Additional file 1: Fig. S1f). In contrast, MethylC-seq and EpiGnome showed early saturation at 2 million reads on 100 pg of input DNA (Fig. 1i). We further conducted a comparison of the LABS on 1 ng and 100 pg of 12 commercial cfDNA samples. Unsurprisingly, the performance of the LABS was only slightly compromised at 100 pg of input cfDNA, with low duplication rate and high genomic coverage observed in both samples (Additional file 1: Fig. S1h, S1i).

### LABS can detect epigenomic abnormality of cfDNA

**Fig. 2:**
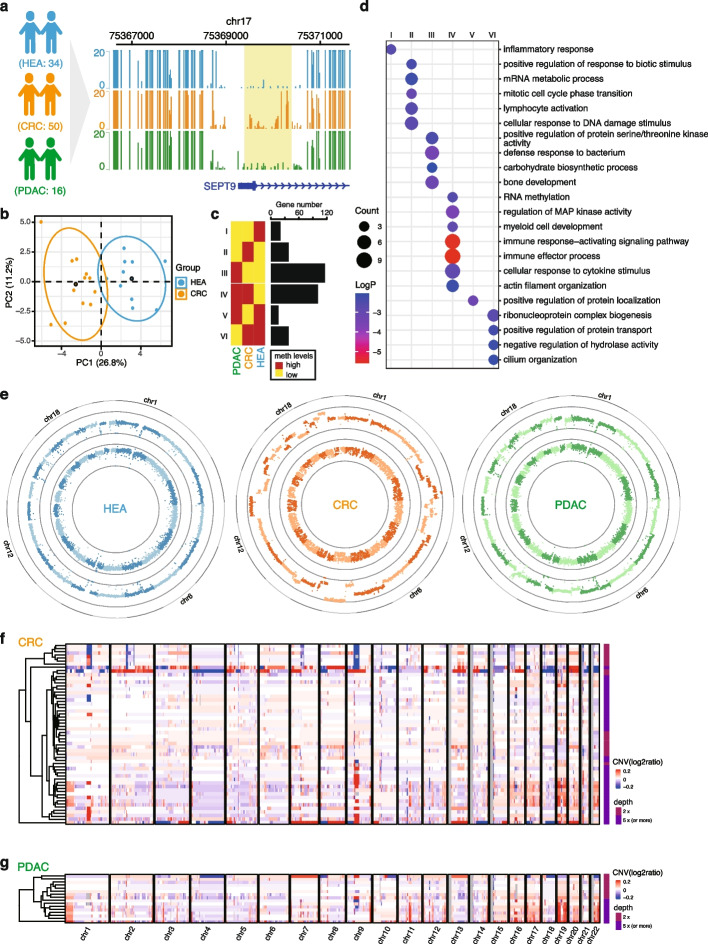
LABS reveals abnormal genomes and epigenomes in cfDNA. **a** Genome browser view of *SEPT9* promoter regions for all 3 groups. The schematic of the clinical experiment design was shown on the left. CRC, colorectal cancer; HEA, healthy controls; PDAC, pancreatic ductal adenocarcinoma. **b** PCA plot showing that deep-sequenced CRC and HEA samples can be separated by methylation levels of promoters of 41 differentially expressed genes from TCGA. **c** Numbers of differentially methylated region-related genes in different combinations of comparisons across the three groups (FDR < 0.01, methylation difference > 15%). **d** Functional enrichment results of differential gene groups from **c**. **e** Circos plots showing the copy number alterations and methylation levels of 1 healthy control sample, 1 CRC sample, and 1 PDAC sample. Inner circles, methylation levels; outer circles, normalized copy numbers. **f** Heatmap showing the normalized copy numbers for all 50 CRC samples. Sequencing depth is shown on the right. **g** Heatmap showing the normalized copy numbers for all 16 PDAC samples. Sequencing depth is shown on the right

We tested the feasibility of using the LABS on clinical cfDNA samples from 50 patients with CRC, 16 patients with PDAC, and 34 race-, age-, gender-matched healthy controls (Fig. 2a, Additional file 1: Fig. S2a, Additional file 3: Table S2). The CRC plasma samples were prospectively collected from consented patients undergoing surgical resections at the University of Chicago Medical Center (UCMC). The PDAC samples were obtained from archived plasma sample collections from patients undergoing the Whipple procedures either at UCMC or at Ochsner Medical Center (provided by City of Hope). The healthy control plasma samples were collected from consented donors undergoing screening colonoscopies at UCMC. After extracting cfDNA from the plasma samples, we generated LABS sequencing libraries using 1 ng of cfDNA per sample (2.5 ng for PDAC) and, on average, sequenced 76.1 million reads per sample.

We observed substantial differences between the CRC and healthy control groups at the *SEPT9* promoter region, the current FDA-approved methylation biomarker for CRC [18] (Fig. 2a), suggesting that the LABS is capable of recapturing this established biomarker. In comparison, the PDAC samples showed low methylation levels in the same region, lending support for the CRC specificity of the *SEPT9* methylated promoter and sensitivity of the LABS.

To further investigate the promoter methylation patterns of known cancer-associated genes, we systematically identified 3188 differentially expressed genes (DEGs) from The Cancer Genome Atlas (TCGA) colon adenocarcinoma (COAD) dataset as colon cancer-associated genes by applying a relatively stringent filtering criterion (FDR (false discovery rate) ≤ 0.05 and logFC (fold change) ≥ 2 or ≤  − 2). Next, we extracted the corresponding promoter regions for these DEGs by flanking 1 kb of each transcription start site (TSS). We then calculated the methylation percentages of these promoter regions in our high-coverage CRC and healthy control samples. Through this process, we identified 41 differentially methylated promoter regions between the CRC and healthy control samples (percent methylation difference ≥ 12%, FDR < 0.01, Additional file 1: Fig. S2d). To further validate the significance of these regions, we utilized them as input to perform a principal components analysis (PCA) and observed that the CRC and healthy control samples were separated based on the methylation levels of these promoter regions, confirming the reliability of promoter DNA methylation based on the LABS data to correlate with at least part of gene expression changes associated with colon cancer (Fig. 2b and Additional file 1: Fig. S2d, S2e).

Next, we identified 1362–10,716 3-kb differentially methylated regions (DMRs) in the genome from cfDNA methylation profiles (Additional file 1: Fig. S2b). Intriguingly, both highly methylated regions specific to the control group and lowly methylated regions specific to CRC and PDAC samples were predominantly localized at the intergenic regions, consistent with what is known as the genome-wide demethylation during tumorigenesis (Additional file 1: Fig. S2b, S2c). In addition, functional enrichment analysis of the DMR-related genes (i.e., host genes with DMRs in their promoters) revealed pathways known to be involved in tumorigenesis in each group (Fig. 2d). For example, the pathway “cellular response to DNA damage stimulus” was enriched in the CRC-specific highly methylated genes, while “carbohydrate biosynthetic process” was enriched in the PDAC-specific highly methylated genes. Importantly, we also observed that “inflammatory response” was enriched in cancer-specific lowly methylated genes for both CRC and PDAC, while “immune response-activating signaling pathways” and “immune effector processes” were enriched in cancer-specific highly methylated genes for both cancer types.

### LABS can detect copy number alternations in cfDNA

During our analysis of the LABS datasets from clinical cfDNA samples, we also observed copy number alterations (CNAs) in cfDNA derived from cancer patients. Standard WGBS methods are prone to PCR amplification bias, resulting in the underrepresentation of DNA fragments (e.g., tumor-derived DNA) that exist in low abundance in the cfDNA. We realized that the linear amplification in our protocol might have additional advantages of retaining DNA fragment composition information of the original, unamplified sample. To test whether we were able to leverage the low coverage bias inherent in linear amplification in order to obtain new information previously unavailable with exponential amplification, we carefully assessed genome-wide copy number alterations in each cfDNA sample. Indeed, we observed substantial chromosome-scale copy number alterations for some patient samples independent of methylation level changes (Fig. 2e). While all healthy controls showed uniform copy ratios, 18/50 CRC and 8/16 PDAC patients showed abnormal copy number alterations (Fig. 2f, g). These identified CNAs from cfDNA were further confirmed as tumor-derived based on previously known CNAs from the TCGA database, with 10 COAD amplification markers and 9 COAD deletion markers found in at least 1 CRC patient cfDNA profile (Additional file 1: Fig. S2f). These results further supported the high accuracy and sensitivity of the LABS on cfDNA.

### LABS can reveal TOO and immune cell composition of cfDNA

Successful application of the LABS to detect CNAs in cancer patient cfDNA prompted us to search for additional information that the LABS could uniquely offer in contrast to traditional assays based on exponential amplification. Of note, circulating cfDNA contains DNA fragments from different tissues and blood cells in the background [19]. Although human tissues possess the same genomic DNA sequence, they have unique DNA methylation patterns [20, 21]. Because the linear amplification nature of the LABS avoids bias introduced by exponential amplification as shown in CNA detection, we proceeded to ask whether we could apply certain algorithms to estimate the relative proportions of TOO sources for DNA fragments or cell origins using the published cell type-specific methylation patterns as reference.

**Fig. 3:**
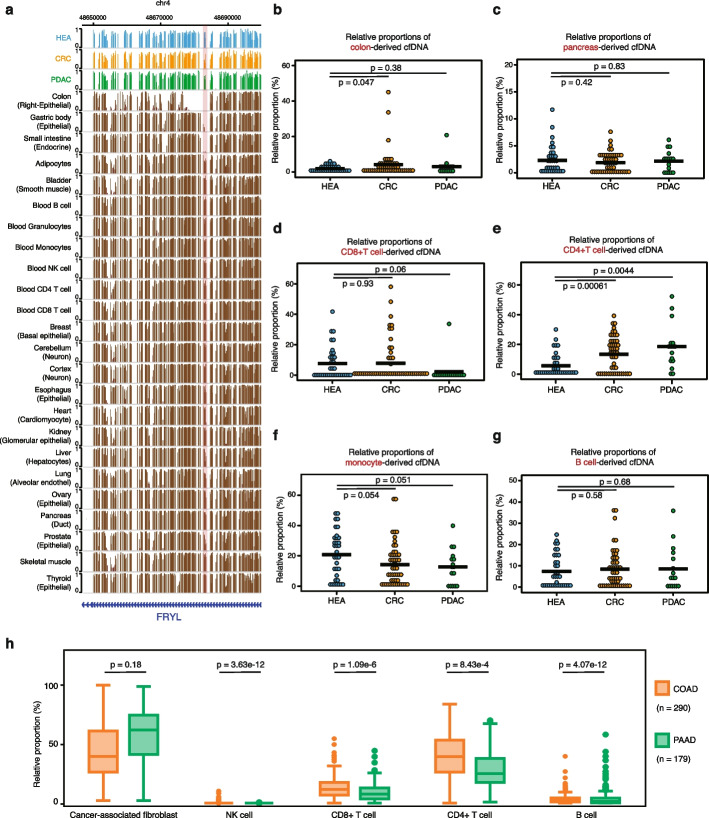
Deconvolution of the LABS profiles reveals tissue-of-origin and immune cell compositions. **a** Genome browser views showing CRC-specific low methylation at colon-specific regions with low methylation. **b** Relative proportions of colon-derived cfDNA in all samples. **c** Relative proportions of pancreas-derived cfDNA in all samples. **d** Relative proportions of CD8 + T cell-derived cfDNA in all samples. **e** Relative proportions of CD4 + T cell-derived cfDNA in all samples. **f** Relative proportions of monocyte-derived cfDNA in all samples. **g** Relative proportions of B cell-derived cfDNA in all samples. **h** Relative proportions of five different cell types from deconvolution of the COAD and PAAD transcriptome datasets in TCGA. COAD, colon adenocarcinoma; PAAD, pancreatic adenocarcinoma; TCGA, The Cancer Genome Atlas

By analyzing the tissue or cell-specific methylation signatures, it is possible to determine the origin of cfDNA fragments using deconvolution algorithms (see the “ Methods” for details). In particular, we found that neutrophils contributed the most to cfDNA (Additional file 1: Fig. S3, S4), consistent with a previous report [22]. As a proof-of-concept, we also observed that both CRC and PDAC patients had a lower percentage of neutrophil-derived cfDNA fragments compared to healthy controls (Wilcox test, *P*-value = 0.059 for CRC and 0.1 for PDAC, Additional file 1: Fig. S4a). Interestingly, the percentages of cfDNA originating from the colon were significantly higher in CRC compared to both healthy controls and PDAC patients (Wilcox test, *P*-value = 0.047), with CRC samples showing low methylation levels at colon-specific, lowly methylated regions (Fig. 3a, b). We did not observe significantly higher levels of pancreas-specific contributions in PDAC-derived cfDNA samples, compared to either healthy controls or CRC patients (Fig. 3c).

Furthermore, functional enrichment results of cancer-specific DMRs showed significant changes in immune-related pathways (Fig. 2d), suggesting the possible existence of distinct immune cell compositions of cfDNA. Results from our immune cell type-specific deconvolution analysis showed notable differences between PDAC samples and healthy controls in their CD8 + T cell proportions, with CD8 + T cells being almost depleted in those PDAC samples (Wilcox test, *P*-value = 0.06, Fig. 3d). This finding is consistent with the immune suppressive features of enriched fibroblast cells in the tumor microenvironment of PDAC. In contrast, the immune cell compositions in CRC samples were more heterogeneous than in PDAC patients. Still, when compared with healthy controls, we observed elevated levels of CD4 + T cells (Wilcox test, *P*-value = 0.00061, Fig. 3e) in CRC samples. Monocytes-derived cfDNA was also decreased in both CRC and PDAC groups (Fig. 3f). We did not find significant differences in B cell-derived cfDNA across the three groups (Fig. 3g). We also applied the similar deconvolution method on the COAD (*n* = 290) and pancreatic adenocarcinoma (PAAD) (*n* = 179) transcriptome datasets from TCGA, further confirming less CD8 + T cell compositions in PAAD samples (Fig. 3h). Overall, our method allowed deconvolution of immune cell types directly from cfDNA methylation analysis and provided insights into the tumor microenvironment in different cancer types when compared to healthy controls (Additional file 1: Fig. S4b).

Finally, we evaluated the performance of the LABS, MethylC-Seq, and EpiGnome methods for immune cell decomposition using the same approach. Our analysis revealed that the LABS consistently produced accurate estimates of different cell types across varying amounts of DNA input (Additional file 1: Fig. S4c). In contrast, MethylC-Seq was unable to detect any T/NK cells (the data range for CD4T + cells are as follows: control 0–23%, MethylC-seq 0–3%, LABS 0–30%, EpiGnome 2–40%; the data range for NK cells are as follows: control 0–28%, MethylC-seq 0%, LABS 0–14%, EpiGnome 0–18%), while EpiGnome overestimated the proportion of eosinophils (the data range for eosinophils are as follows: control 0–51%, MethylC-seq 5–100%, LABS 0–30%, EpiGnome 32–50%). These discrepancies in accuracy were likely caused by their nonlinear amplification approach, which is known to introduce bias into the final amplified products.

### A multi-faceted approach to increase the accuracy of CRC detection using cfDNA

**Fig. 4:**
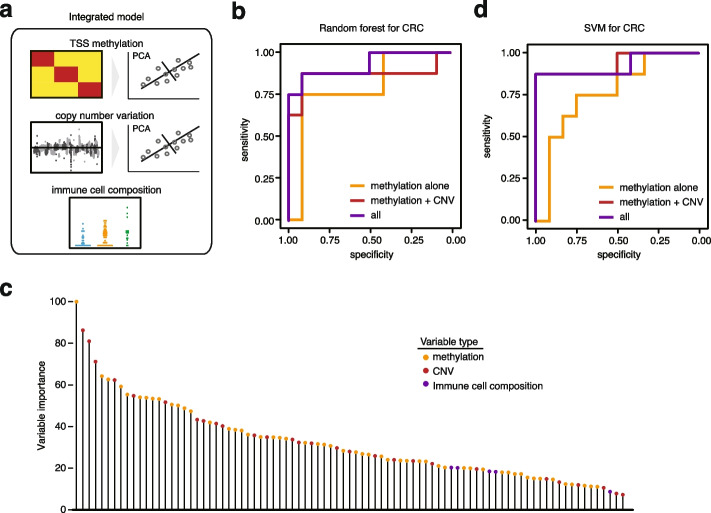
Integrating multiple layers of information from the LABS provides a better prediction. **a** Diagram of the integrated model. TSS methylation and copy number variations were first analyzed by PCA to find the most informative PCs. **b** ROC curves showing better prediction accuracy of the integrated random forest model for CRC compared to methylation biomarkers alone. CNV, copy number variation; ROC curve, receiver operating characteristic curve. **c** Variable importance of the random forest model shown in **b**. Each bar represents a variable, i.e., a principal component based on TSS methylation/a copy number variant, or the relative percentage of a particular immune cell type. **d** ROC curves showing better prediction accuracy of the integrated SVM model for CRC compared to methylation biomarkers alone. SVM, support vector machine

We evaluated whether an integrated model that combined multiple types of features obtained from the LABS could provide higher predictive accuracy than methylation biomarkers alone (Fig. 4a), using CRC samples, with which we had a relatively larger sample size. Specifically, we randomly divided the 84 samples (comprising CRC and healthy individuals) into a training set and a test set, in a 1:3 ratio. We obtained 3 types of data: methylation, copy number, and immune cellular compositions, and utilized both random forest and support vector machine (SVM) to build classifiers. We initially tested methylation features within TSS regions alone and then added copy number ratios and immune cell compositions to evaluate the performance of the integrated model. For methylation features and copy number ratios, we conducted PCA to reduce dimensions. All features were rescaled and standardized (details provided in the “ Methods” section).

In the testing samples (*n* = 84), our findings indicated that using TSS methylation alone, the random forest algorithm achieved an area under the curve (AUC) of 0.79. However, by incorporating copy number data, the AUC increased to 0.91, and the further inclusion of immune cell proportions resulted in an AUC of 0.93 (Fig. 4b). Conversely, training on immune cell proportions alone and copy number alone yielded lower AUCs of 0.59 and 0.88, respectively. Similarly, SVM produced comparable results (Fig. 4d). Notably, the most significant feature identified by both random forest and SVM was a principal component generated from the methylation data (Fig. 4c and Additional file 1: Fig. S5b), including TSS methylation of *FGFR1*, *MYO6*, and *CDK9*, among others. Functionally, high expression of *FGFR1* has been shown to be associated with increased proliferation and invasion of colorectal cancer cells [23]; *MYO6* is known to be an oncogene in CRC [24], while *CDK9* has been used as a potential target for the treatment of CRC [25]. Overall, our analysis indicated that combining multiple feature types obtained from the LABS could lead to higher detection accuracy than methylation biomarkers alone.

## Discussion

Although cfDNA analysis holds great potential for non-invasive early cancer detection, there are still obstacles to overcome before broad applications in “real-world” clinical settings. A major challenge is the variation in the amount of ctDNA present in different samples, which can range from less than 0.01% to over 90% of total cfDNA [2]. This variability affects the sensitivity of the assay and ultimately impacts its clinical utility. The value of liquid biopsies for cancer detection is therefore contingent on our ability to detect ctDNA within a larger amount of background DNA. Our LABS method utilizes T7 RNA polymerase-based in vitro transcription to generate single-stranded DNA fragments after bisulfite treatment, preserving genomic information in a lineal and unbiased fashion of amplification. This strategy avoids potential amplification bias that is typically associated with exponential amplification approaches and is particularly suitable for cfDNA as the short lengths of cfDNA fragments may not be efficiently amplified by multiple displacement amplification type approaches. As a result, the LABS is shown to achieve high genomic coverage and low coverage biases using sub-nanogram input DNA, representing a significantly improved DNA methylation profiling technique that will benefit cfDNA-based cancer detection and screening, for example, featuring with ~ 20-fold increased sensitivity when compared with MethylC-Seq and EpiGnome methods at 0.1 ng of input DNA.

Moreover, the high genomic coverage and unbiased amplification provided by the LABS allows for a wide range of analyses to be performed which were not previously possible. The linear amplification of all cfDNA fragments preserves underrepresented cfDNA components and allows sensitive detection of methylation features especially from ctDNA. The methylation signals within the promoter regions of ctDNA are well-known predictors of gene expression, allowing elucidation of functional pathways associated with the tumor. Secondly, the linear amplification of our LABS method enables sensitive detection of copy number alterations even from single cells [26], making CNA detection possible from limited input of cfDNA.

Importantly, we demonstrated that the LABS could be used for cellular deconvolution in order to reveal the TOO of cfDNA sources and immune cell composition from the original, unamplified cfDNA. The unique methylation patterns of specific cell types have long been used to classify different cell or tissue types. The abilities of the LABS data to be used to deconvolute and assess relative levels of cfDNA fragments derived from different cell types based on methylation signatures could open unprecedented opportunities. As we showed, approximately 50% of cfDNA in cfDNA samples was derived from neutrophils, with the remainder coming from other cell and tissue types. With the ability to identify the origin of cells at the read level and examine tissue-specific methylation signatures of different cfDNA fragments, we may be able to reduce background noise and improve the accuracy of cancer detection. This new method allows us to detect and assess levels of not only ctDNA from human cancers but also cfDNA sources that represent altered immune cell composition or tumor-adjacent tissues. We showed that the deconvolution of immune cells-derived cfDNAs from PDAC samples resulted in lower percentages of CD8 + T cell component, consistent with the immune-resistant characteristics of PDAC. The levels of cfDNA components derived from CD4 + T cells and intestinal cells detected by the LABS could be promising markers themselves to enhance the sensitivity and specificity for colon cancer detection and screening. Sensitive detection and assessment of multiple features encoded in cfDNA revealed by the LABS can thus provide tumor and immune cell status simultaneously, showing the promise to be assessed for prognosis and/or guiding immunotherapies as well in the future.

In addition, the same approach as described in the current work can be applied to a wide range of human diseases beyond cancers. Circulating cfDNA from diseased cells, adjacent tissues, and immune cells could be used to tease apart pathogenic mechanisms underlying these disorders. A promising avenue for future research is to further develop machine learning and artificial intelligence (AI)-based models that can separate reads based on the origins of their DNA fragments in cfDNA.

## Conclusions

The LABS is a highly sensitive and effective method for methylation profiling, copy number aberration detection, and cell type deconvolution using amounts of input DNA feasible from clinical biospecimens. By extending information beyond differential methylation sites or regions, the LABS may significantly improve the power of methylation-based cfDNA analysis, thus helping to establish liquid biopsies as a standard tool in health care in the future.

## Methods

### Cell culture and genomic DNA isolation

The mouse embryonic stem cells (mESC) were grown on gelatin-coated plates in Dulbecco’s modified Eagle medium (DMEM) (Invitrogen Cat. No. 11995) supplemented with 15% FBS (Gibco), 2 mM l-glutamine (Gibco), 1X nonessential amino acids (Gibco), 1% penicillin/streptavidin (Gibco), 1 × β-mercaptoethanol (Sigma), 1000 u/mL leukemia inhibitory factor (Millipore Cat. No. ESG1107), 1 μM PD0325901 (Stemgent, dissolved in DMSO), and 3 μM CHIR99021 (Stemgent, dissolved in DMSO). All cells were cultured at 37 °C under 5.0% CO_2_ and passaged every 2 days. For genomic DNA isolation, cells were harvested by centrifugation for 3 min at 1000 × *g*. DNA was extracted with the AllPrep DNA/RNA Mini Kit (Qiagen) according to the manufacturer’s protocol.

### Patient recruitment and collection of plasma samples

A total of 50 patients with colorectal cancer (CRC), 15 patients with pancreatic ductal adenocarcinoma (PDAC), and 34 healthy individuals were recruited at the University of Chicago Medical Center. One additional PDAC patient sample was supplied by the City of Hope, with original patient consent and collection at Ochsner Medical Center in New Orleans. Blood from patients with CRC and PDAC was collected prior to surgical resection (CRC), Whipple treatment (PDAC), and adjuvant chemotherapy or other radical treatments. Blood from healthy individuals was collected from those individuals who underwent screening colonoscopies at the University of Chicago Medical Center with no malignant diseases nor advanced adenomas found upon post-colonoscopy pathological analyses. Blood samples were collected in K_2_ EDTA or Streck vacutainers and were centrifuged for 1350 × *g* for 12 min at 4 °C twice, and 13,500 × *g* for 5 min at 4 °C. The plasma fraction was reserved and stored at − 80 °C until cfDNA extraction. Circulating cfDNA was isolated from 0.3–2 mL plasma using the QIAamp Circulating Nucleic Acid Kit (Qiagen). CfDNA was quantified with Qubit. The informed consent form was obtained from each study participant. All patients were consented under IRB-10–209 A. The protocol is approved by the University of Chicago Institutional Review Board.

### Preparation of T7 adaptors

Oligos for the T7 sequence /5Phos/iMe-dC/iMe-dC/iMe-dC/TATAGTGAGT/iMe-dC/GTATTAATTT/iMe-dC/G/iMe-dC/GGGG/iMe-dC/T and short helper CGACTCACTATAGGGT/3Phos/ were dissolved in annealing buffer (10 mM Tris–HCl pH 8.0, 0.1 mM EDTA, 50 mM NaCl). The T7 adaptors were prepared by mixing the two oligos equally to 50 μM and were annealed using a PCR machine (95 °C 5 min, − 0.25 °C/min cooling down to 4 °C). The adaptors were diluted to 15 μM with the annealing buffer and stored at − 20 °C.

### Bisulfite conversion

Bisulfite treatment was carried out on fragmented gDNA or cfDNA using the MethylCodeTM Bisulfite Conversion Kit (Invitrogen, MECOV50), producing 9–20 μL eluate in nuclease-free H_2_O.

### MethylC-seq WGBS library preparation

The mESC gDNA was fragmented into 150–400-bp dsDNA fragments. Sequencing libraries were prepared using the NEXTflex® Bisulfite Sequencing Kit (PerkinElmer) according to the manufacturer’s protocol. Briefly, after end repair and 3′-adenylation reaction, the methylated adaptor was ligated to two ends of DNA fragments. Then, DNA was subjected to bisulfite conversion. Finally, the library was amplified using the KAPA Hifi Uracil Plus Polymerase (Kapa Biosystems) and purified by 0.8X AMPure XP beads twice. The library was sequenced using the NextSeq 500 SR80 platform at the University of Chicago Genomics Core Facility.

### Epignome WGBS library preparation

The TruSeq DNA Methylation Kit (Illumina, Inc., San Diego, USA) was used. gDNA or cfDNA was subjected to bisulfite conversion at first. Synthesis random primers were annealed to converted ssDNA and following the manufacturer’s protocol. DNA strands containing a specific sequence tag from random primers were synthesized. Then, a known sequence tag was added to the 3′-end of DNA strands. The di-tagged DNA was purified by using 1.6X AMPure XP beads. The library was amplified by the Failsafe PCR enzyme system and cleaned up with 1.0X AMPure XP beads. The library was sequenced with the NextSeq 500 SR80 platform at the University of Chicago Genomics Core Facility.

### LABS library preparation

Extracted cfDNA or fragmented gDNA was ligated with T7 adaptor using KAPA Hyper Prep Kit (Roche Cat. No. KK8504). Briefly, DNA in nuclease-free H_2_O was mixed with end repair and A-tailing buffer and end repair and A-tailing enzyme mix and incubated at 20 °C 30 min and 65 °C 30 min. Next, the homemade T7 adaptor, H_2_O, ligation buffer, and DNA ligase were added and incubated at 20 °C for 4 h or 4 °C overnight. After post-ligation clean-up using 1.4X AMPure XP Beads, DNA was subjected to bisulfite treatment. Bisulfite-converted DNA was extended via using EpiMark Hot Start Taq DNA Polymerase (NEB, M0490S) with T7 Primer (AGCCCCGCGAAATTAATACGACTCACTATAGGG, IDT with HPLC purification). Qiagen protease (Qiagen, Cat. No. 19155) was added and incubated at 50 °C for 2 h, followed by heat inactivation at 75 °C for 30 min. The T7-tagged dsDNA fragments were used as a template to generate in vitro transcription via HiScribe™ T7 High Yield RNA Synthesis Kit (NEB, E2040S) and SUPERase•In™ RNase Inhibitor (Life Technologies, AM2694) and were added in the reaction. The reaction was incubated at 37 °C for 12–16 h.

After overnight T7 in vitro transcription, DNase I (NEB, M0303S) and digestion buffer were added to digest DNA templates at 37 °C for 10 min. Then, RNA transcripts were purified by RNA Clean and Concentrator kit (Zymo Research, R1013). RNA yield was quantified by Qubit 2.0 RNA HS Assay Kit (Life Technologies, Q32855). A maximum of 100 ng RNA was used for library construction (Roche, KAPA RNA HyperPrep kit, KK8541) following the manufacturer’s protocol. Libraries were sequenced at the University of Chicago Genomics Core Facility.

### Processing of the LABS data

Sequencing adaptors and low-quality nucleotides were trimmed from raw sequencing reads by Trim_Galore and then aligned to the human reference genome (hg19) by Bismark according to a published processing procedure. Further duplication removal and methylation calling were performed by scripts of the Bismark package. The methylation proportion of each CpG (at least 3 ×) was determined using the methylKit R package. Besides CpG-level data, for analytical convenience, data was also summarized into unbiased, genome-wide 3-kb sliding windows for downstream analyses, represented by the weighted mean of CpGs for each bin. Differential methylation regions were detected by methylKit R package, with a cutoff of FDR < 0.01 and methylation differences larger than 15%. Metagene plot and Lorenz curve were generated by deepTools. GC content bias was assessed by Picard tools. IGV was used for the genome browser view. To avoid comparison bias by different sequencing depths, we down-sampled 15 million uniquely mapped reads for each sample for methods comparison. Assignment of CpGs to different genomic features (e.g., exons, introns) was done by Homer with a default priority list.

### Copy number detection

For copy number alteration analysis, we keep all sites with 1 × coverage. We used bedtools to extract counts for each genomics bin of 100 kb in hg19 for each sample from its bam file. Then, we pooled together counts for all normal samples as a panel of synthetic controls. We used SynthEx (v1.0.5) to compute the copy ratio for each cancer sample.

### Tissue-of-origin and cell type-specific deconvolution

For TOO and cell type-specific deconvolution analysis, we keep all sites with 1 × coverage. For tissue-of-origin analysis, 1013 previously curated markers were used to identify the tissue-of-origin for cfDNA [22]. These markers represent genomic regions that exhibit a methylation level that is significantly different in 1 tissue compared to others. The methylation level for each genomic region in each sample was estimated by calculating the proportion of methylated reads that cover all sites within the same group. To address missing values in our dataset, we applied the *impute.knn* function from R. Next, we used EpiDISH [27] to perform deconvolution analysis with the Cibersort-CBS method.

For immune cell deconvolution, we used a similar approach to the tissue-of-origin analysis. We selected 333 CpG sites from PBMC samples as reference sites for immune cell deconvolution using EpiDISH [27]. Detecting all reference sites in a single LABS data sample can be difficult due to coverage insufficiency. To overcome this issue, the reference sites were split by dendrogram using the *cutree* function into 32 groups. The sites within each group were then aggregated to form a mega site, and singleton clusters were removed, resulting in a final set of 26 mega sites. Each mega site consists of 2 to 50 CpG sites. Missing values were imputed by the *impute.knn* function. We then used these mega sites to perform cell type deconvolution using EpiDISH with the Cibersort-CBS method. For statistical analysis, we employed the *t.test* function.

### Binary classification of CRC and healthy controls

We randomly divided the 84 samples (comprising CRC and healthy individuals) into a training set and a test set, in a 1:3 ratio. We obtained three types of data: methylation, copy number, and immune cellular compositions. We preprocessed these data separately.

For methylation data, we computed beta values for 30,984 TSS regions (flanking 1 kb of each TSS) based on GENCODE annotations. We standardized and normalized the methylation training data using the *preProcess* function from the caret package. Next, we performed principal component analysis (PCA) on the standardized and normalized training data using the *prcomp* function in the R Statistical Environment [28] to reduce the dimensionality of the data. We retained 54 principal components (PCs) based on the cumulative proportion of variance explained by the components (95%). We obtained the reduced training data by taking the principal components from the PCA output, and we applied the PCA transformation to the test set.

For copy number data, we used the log_2_ copy ratios within each 100-kb genomic bin. In total, we had 28,823 100-kb genomic bins in the hg19 genome. We removed any bin with “NA” values, leading to a list of 26,903 bins. We performed rescaling and PCA on copy number data, resulting in 28 PCs as input features.

To limit collinearity, we excluded one cell type (i.e., eosinophils) from the immune cellular compositions data, after which the proportion data of the remaining six immune types were standardized and normalized.

The methylation, copy number, and proportion data were combined for the training and test sets. We trained a random forest model on the training data using the *train* function in caret with fivefold cross-validation. The model’s performance was evaluated on the test set using the *predict* function, and the ROC curve was plotted using the *roc* and *plot* functions from the pROC package.

The variable importance of the trained model was visualized using the *varImp* and *plot* functions in caret. Additionally, we used the *train* function in caret to train a support vector machine model using a radial kernel. The recursive feature elimination implemented in the kernlab package was used to assess feature importance for SVM. We also trained models using only methylation or copy number data, using the same settings.

### Supplementary Information

Supplementary Material 1:  Fig. S1. Validation of the LABS. Fig. S2. The LABS simultaneously detects differentially methylated regions and copy number alterations in CRC and PDAC samples. Fig. S3. Genome browser view showing high consistency of cfDNA profiles with blood granulocytes profiles at granulocyte-specific regions with low methylation. Fig. S4. Deconvolution of the LABS profiles reveals component-level differences in different groups. Fig. S5. Integrating multiple layers of information from the LABS provides a better prediction based on SVM.

Supplementary Material: Table S1. Sequencing information of all samples for the method comparisons.

Supplementary Material 3: Table S2. Demographical and clinical information of the study participants.

Supplementary Material 4: Review history.

## Data Availability

The dataset supporting the conclusions of this article is available in the NCBI Gene Expression Omnibus (GEO) repository, GSE186007 [29]. All codes used in this study have been deposited into the GitHub repository under the MIT License (https://github.com/xlcui/LABS) [30] and also into Zenodo (https://doi.org/10.5281/zenodo.11061161) [31].
