# Supplementary material for: LABS: linear amplification-based bisulfite sequencing for ultrasensitive cancer detection from cell-free DNA
Source: Genome Biol. 2024 Jun 14;25:157. doi: 10.1186/s13059-024-03262-2 (PMC11177480; doi:10.1186/s13059-024-03262-2)
Supplement: Supplementary file 1 — Supplementary Material 1: Fig. S1. Validation of the LABS. Fig. S2. The LABS simultaneously detects differentially methylated regions and copy number alterations in CRC and PDAC samples. Fig. S3. Genome browser view showing high consistency of cfDNA profiles with blood granulocytes profiles at granulocyte-specific regions with low methylation. Fig. S4. Deconvolution of the LABS profiles reveals component-level differences in different groups. Fig. S5. Integrating multiple layers of information from the LABS provides a better prediction based on SVM. [file 13059_2024_3262_MOESM1_ESM.docx]

**Supplementary Information**

**LABS: linear amplification-based bisulfite sequencing for ultrasensitive cancer detection from cell-free DNA**

Xiao-Long Cui^1,2,3 †^, Ji Nie^1,2 †^, Houxiang Zhu^4,5 †^, Krissana Kowitwanich^1,2^, Alana V. Beadell^1,2^, Diana C West-Szymanski^1,2,4^, Zhou Zhang^3^, Urszula Dougherty^4^, Akushika Kwesi^4^, Zifeng Deng^4^, Yan Li^4,5^, Danqing Meng^6^, Kevin Roggin^7^, Teresa Barry^4^, Ryan Owyang^1,2^, Ben Fefferman^1,2^, Chang Zeng^3^, Lu Gao^1,2,4^, Carolyn WT Zhao^1,2^, Yuri Malina^1,2^, Jiangbo Wei^1,2^, Melanie Weigert^8^, Wenjun Kang^4,5^, Ajay Goel^9^, Brian C.-H. Chiu^10^, Marc Bissonnette^4^, Wei Zhang^3*^, Mengjie Chen^4,5*^, Chuan He^1,2*^

^1^Department of Chemistry, Department of Biochemistry and Molecular Biology, Institute for Biophysical Dynamics, University of Chicago, Chicago, IL, USA

^2^Howard Hughes Medical Institute, University of Chicago, Chicago, IL, USA

^3^Department of Preventive Medicine, Northwestern University Feinberg School of Medicine, Chicago, IL, USA

^4^Department of Medicine, The University of Chicago, Chicago, IL, USA

^5^Department of Human Genetics, The University of Chicago, Chicago, IL, USA

^6^Department of Neurobiology, Northwestern University, Evanston, IL, USA

^7^Department of Surgery, University of Chicago, Chicago, IL, USA

^8^Department of Obstetrics and Gynecology/Section of Gynecologic Oncology, University of Chicago, Chicago, IL, USA

^9^City of Hope Comprehensive Cancer Center, Duarte, CA, USA

^10^Department of Public Health Sciences, The University of Chicago, Chicago, IL, USA

^†^These authors contributed equally: Xiao-Long Cui, Ji Nie, Houxiang Zhu.

*These authors jointly supervised this work: Chuan He ([chuanhe@uchicago.edu](mailto:chuanhe@uchicago.edu)), Mengjie Chen ([mengjiechen@uchicago.edu](mailto:mengjiechen@uchicago.edu)), or Wei Zhang ([wei.zhang1@northwestern.edu](mailto:wei.zhang1@northwestern.edu)).


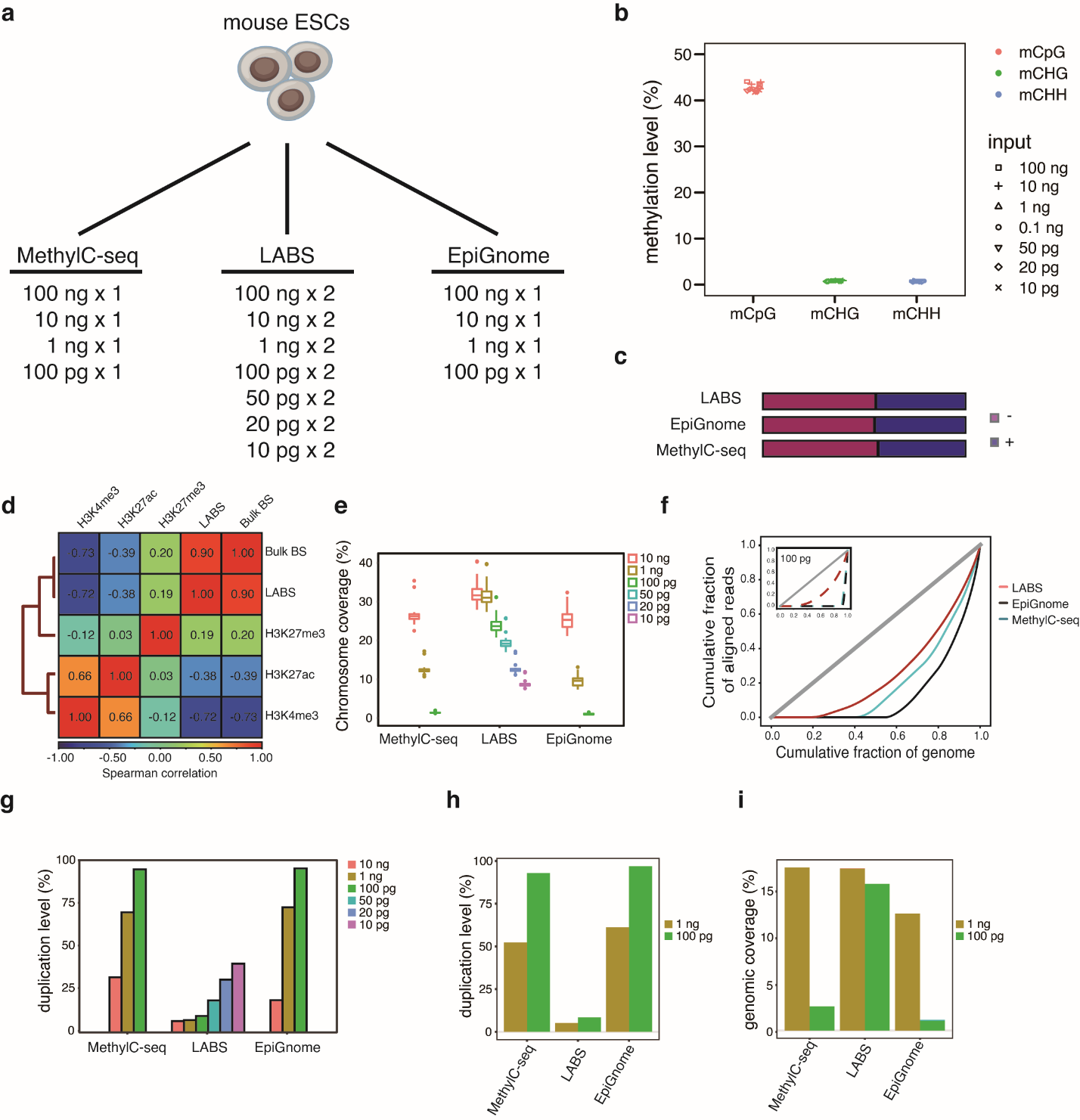


**Fig. S1. Validation of the LABS. a** Schematic plot of the experimental design. Genomic DNA from mouse ESCs was used to build libraries using three different methods and various input DNA amounts. **b** Cytosine methylation levels of different contexts. **c** Strand distribution of sequencing reads from all three methods. **d** Spearman correlation coefficients of paired comparisons between LABS, bulk WGBS, H3K27me3, K3K27ac, and H3K4me3. **e** Chromosome coverages for all three methods from different input DNA amounts. **f** Lorenz curves showing less biased reads distributions of LABS compared to the other two methods from 10 ng input DNA. The insert figure shows the same curves from 100 pg input DNA. **g** Duplication levels for all three methods from different input DNA amounts. **h** Duplication levels for all three methods from different input cfDNA amounts. **i** Genomic coverages for all three methods from different input cfDNA amounts.


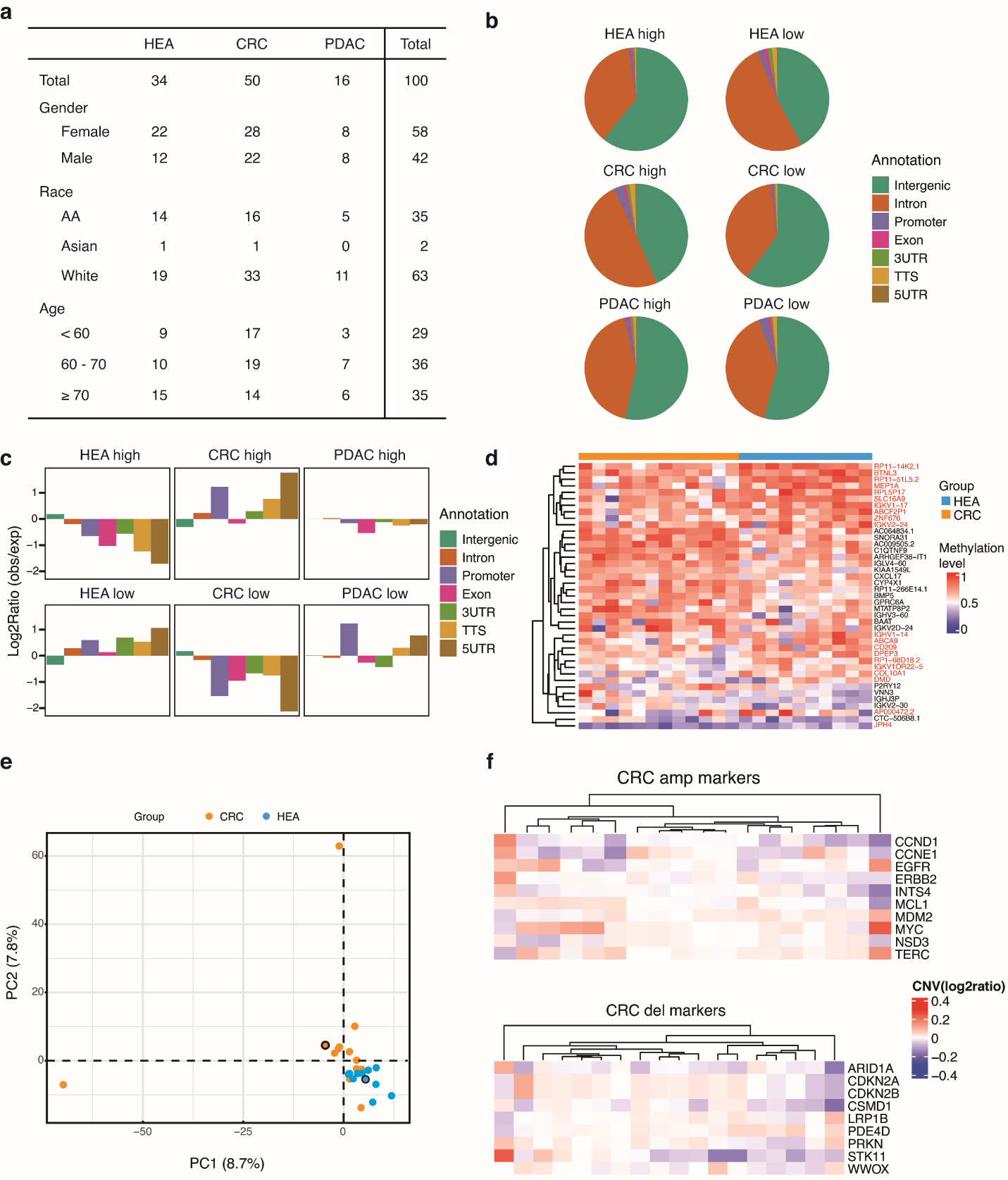


**Fig. S2. LABS simultaneously detects differentially methylated regions and copy number alterations in CRC and PDAC samples. a** Demographic characterization of all 100 samples. **b** Genomic distributions of group specific differentially methylated regions (DMRs). HEA high, DMRs specifically highly methylated in healthy control group; HEA low, DMRs specifically lowly methylated in healthy control group; CRC high, DMRs specifically highly methylated in CRC group; CRC low, DMRs specifically lowly methylated in CRC group; PDAC high, DMRs specifically highly methylated in PDAC group; PDAC low, DMRs specifically lowly methylated in PDAC group.  **c** Enrichment of group specific DMRs on indicated genomic elements. Observation to expectation ratios are shown. **d** Methylation levels of top differentially expressed genes from TCGA COAD dataset in deep sequenced cfDNA samples. Genes with upregulated expression in cancer samples are labeled in red, and genes with downregulated expression in cancer samples are labeled in black. **e** PCA plot showing that deep sequenced CRC and HEA samples can be partly separated by methylation levels of promoters of 3188 differentially expressed genes from TCGA. **f** Copy number alterations of CRC samples on known amplification and deletion markers from TCGA database.

**
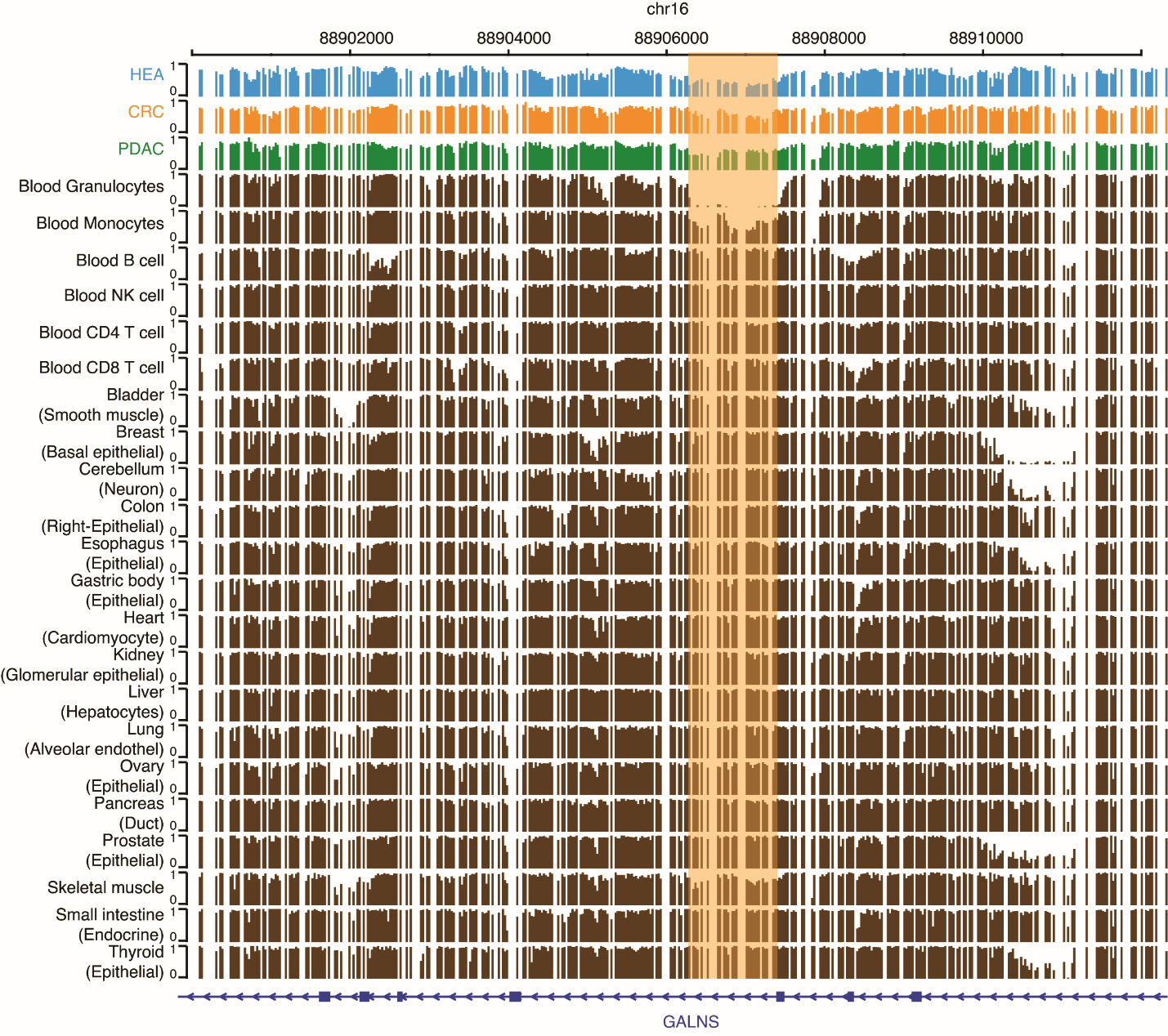
**

**Fig. S3. Genome browser view showing high consistency of cfDNA profiles with blood granulocytes profiles at granulocyte-specific regions with low methylation.**


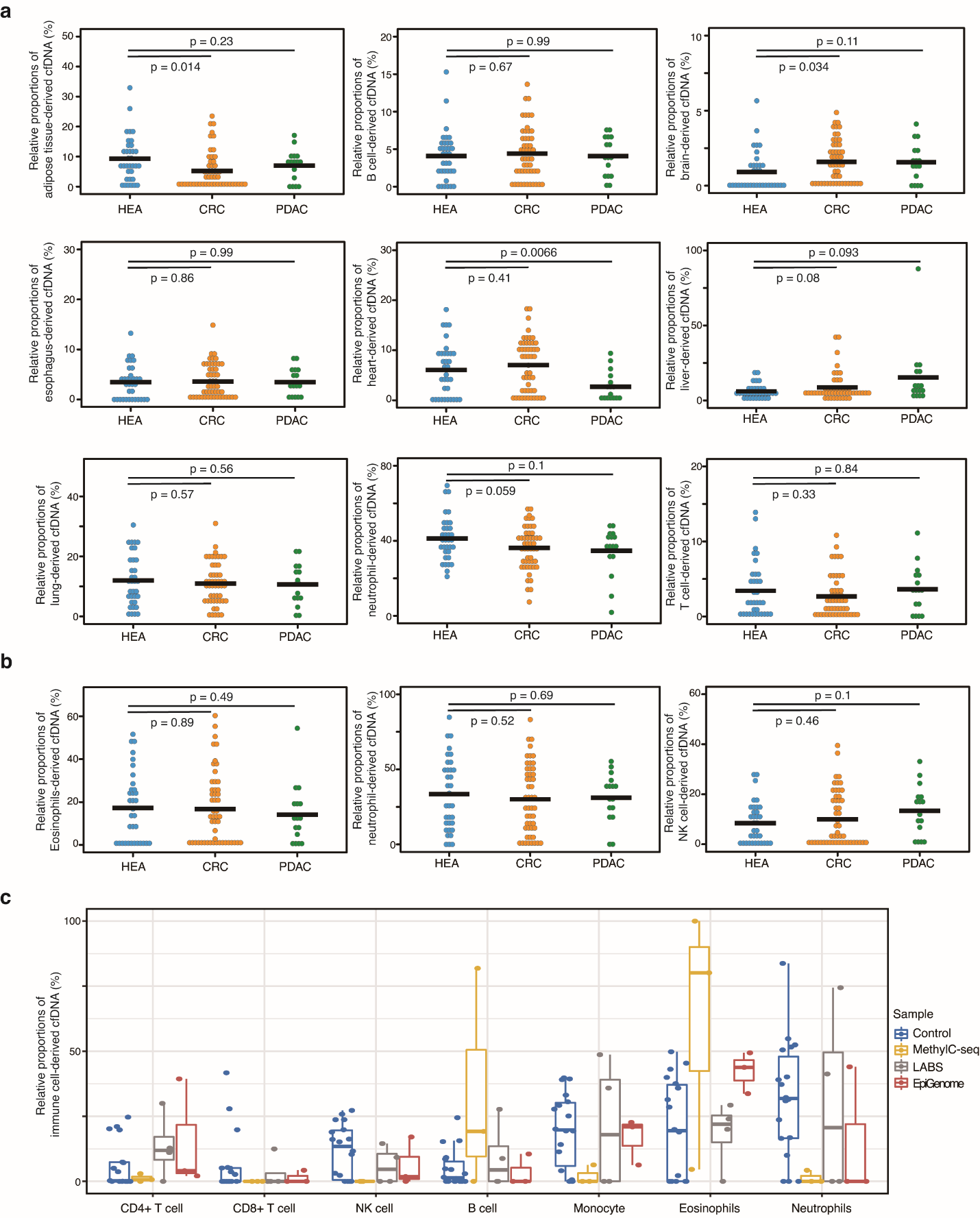


**Fig. S4. Deconvolution of the LABS profiles reveals component-level differences in different groups. a** Deconvolution results of 100 LABS profiles based on 14 reference tissues and cell types. **b** Immune cell type specific deconvolution of 100 LABS profiles based on 7 reference cell types. **c** Immune cell type specific deconvolution of cfDNA samples using three different library construction strategies on commercial cfDNA samples from healthy individuals, as shown in Additional file 2: Table S1. Control, deconvolution results from healthy samples profiled by the LABS as shown in Fig. 2a.


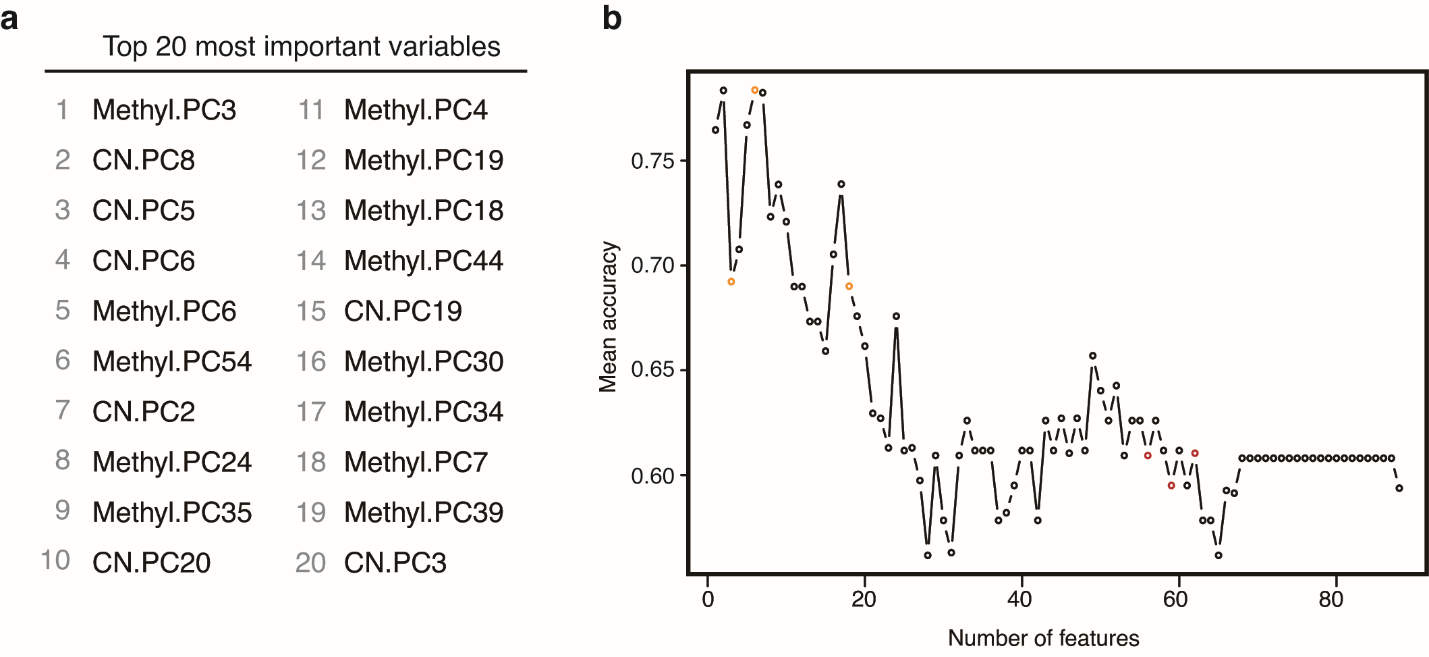


**Fig. S5. Integrating multiple layers of information from the LABS gives a better prediction based on SVM. a** Top 20 most important variables in the random forest model. PC, principal component. Methyl, TSS methylation. CN, copy number ratios. **b** Recursive feature elimination results in the integrated SVM model.

**Table S1: Sequencing information of all samples for the method comparisons.**

**Table S2: Demographical and clinical information of the study participants.**
