# Supplementary material for: LABS: linear amplification-based bisulfite sequencing for ultrasensitive cancer detection from cell-free DNA
Source: Genome Biol. 2024 Jun 14;25:157. doi: 10.1186/s13059-024-03262-2 (PMC11177480; doi:10.1186/s13059-024-03262-2)
Supplement: Supplementary file 4 — Supplementary Material 4: Review history. [file 13059_2024_3262_MOESM4_ESM.docx]

**1^st^ round**

**Reviewer 1**

In the article "Linear Amplification based Bisulfite Sequencing Enables Ultrasensitive Cancer Detection from Cell-free DNA", the authors describe a new method entitled LABS to detect cancer from cell-free DNA from blood using the detected abnormalities in DNA methylation levels. The paper is well-written, the results are convincing and address an important challenge in the field. There are just few minor points that need to be addressed.

Major comments

1. The authors make a PCA (fig. 2b) using differentially methylated regions, i.e pre-selecting features that are differential between the two groups. It would be good to see how the PCA looks like if taking the methylation of all TSS from differentially expressed genes. If this does not show the separation in groups it would be good to nuance the statements, confirming the reliability of DNA methylation to correlate with part of the GE changes. Important to note is that all the data can only talk about correlations not causal predictions.

2. Related to the remarks "We did not find significant differences in B cell-derived cfDNA among the three groups (Fig. 3d). This finding is consistent with the immune suppressive features of enriched fibroblast cells in the tumor microenvironment of PDAC." It is not clear how the second sentence follows from the first one, this could be explained better, e.g. how does the lack of difference in B-cells relate to immune suppression?

3. It is unclear how suppl. fig 4c should be interpreted. What does "health" mean and how is it computed. And the different fractions using the other methods, do they come from healthy samples? And from which amount of input DNA. It seems also that the link to suppl. fig 4c is missing in the text.

Minor comments

4. Figures:
- Legend in 1j is missing. Though the legend is the same as 1i, it would be nice to repeat it, or put it completely to the left, to see that the legend indeed belongs to i and j.
- Suppl. Figure 1i seems to miss a legend.
- "Coverages on both DNA strands were comparable for all three methods (Supplementary Fig. 2c)." I think the authors aim to refer to suppl. fig 1c here.
- Figure 4c is difficult to follow. Why are there mutliple entries per layers, maybe the legend or the figure could be made more explanatory (Suppl. fig 5 for example is more explanatory in this sense)
- It would be informative in suppl figure 2d to label which genes are differentially up- and which are downregulated.

5. Abbreviations: At page 8 it would be maybe good to introduce again the abbreviations PDAC and CRC for the readers that are less familiar with these terms. Same holds true for COAD and PAAD elsewhere in the result section of the manuscript.

6. I tried to find how CpGs were assigned to genomic context (CDS, intron, UTR etc.) but I think it is not listed in the methods, could it be added?

**Reviewer 2**

The manuscript by Cui et at. applied linear amplification, previously developed for single-cell DNA sequencing (e.g. in LIANTI), to DNA methylation sequencing by combining it with bisulfite sequencing. Linear amplification is known to reduce PCR bias and has more even genome coverage. The authors showed the resulting Linear Amplification based Bisulfite Sequencing (LABS) could also reduce the bias in DNA methylation detection compared to traditional PCR-based bisulfite sequencing. The authors then mainly focused on cell-free DNA (cfDNA) application, and showed LABS can be applied to low input (1-2.5 ng) cfDNA to provide multiple features including cancer-specific methylation patterns, copy number alterations, and tissue deconvolution for cancer detection.
Comments:
1. There are numerous cfDNA bisulfite sequencing studies showed the capability to detect CNVs and perform tissue deconvolution. Without more direct head-to-head comparison, it may be premature to say LABS is much better at CNV detection and tissue deconvolution. For example, for those CNVs detected in CRC and PDAC, could normal bisulfite sequencing also detect them? On the other hand, is there any normal cfDNA sequencing done as positive control to show these are indeed CNVs? If the authors break down cancer by stages, do late stage cancers have more CNVs?
2. Fragmentation profile is another important feature of cfDNA. The unbiased LABS should mean it better preserve the fragmentation profile of cfDNA than standard bisulfite. Have the authors looked into that?
3. On the cancer diagnosis part, how is the model performance in different cancer stages? The authors only showed the model for CRC. What about PDAC detection? Since LABS can detect tissue of origin, could it detect CRC and PDAC simultaneously?
4. Figure 1j, what is the reason for the peak around 60% GC percentage in LABS? What would standard DNA sequencing looks like?
5. What is the mESCs methylation level detected by EpiGenome and MethylC-seq?
6. Supplementary Fig. 2d, I cannot see a difference between healthy and CRC.
7. Line 270-275, is it talking about Supplementary Fig. 4c, which was never cited in the main text? It's unclear what data is this. Are MethylC-seq and EpiGenome been applied to the same cfDNA cohort as LABS?
8. Some figures are cited wrong. Line 165, Supplementary Fig. 2c should be Supplementary Fig. 1c. Line 290, Supplementary Fig. 5b should be Fig. 4d.

**Reviewer 3**

Prof Chuan He and colleagues applied their published linear amplification method in another low-input material field, namly cfDNA studies, here also combined with methylation detection. Whereas less-biased amplification is probably advantageous in many settings, I have some concerns about the study design and the strategy that is used to proof this point.

Major concerns:
1. Data is presented that show that the assay works on minute amounts of DNA, which is impressive, but not as relevant to the field as discussed here - Normally, 10ng of cfDNA is available from an average blood draw. The far more important question is, if the method is able to detect very early stages or minimal residual disease to be helpful for screening approaches or therapy monitoring. To test sensitivity and specificity of the approach, linear tests with spike-in probes (e.g. with specific genotypes) should be used to proof the advantages from linear amplification over conventionals PCR approaches and on cfDNA, and not cell lines (l 119ff, 140ff). The coverage of CpGs is indeed impressive compared to the other two assays, but it needs to be shown in the proposed context.
2. the establishment of biomarkers in a targeted approach would put this study to another level
3. the immune cell composition in cfDNA is heavily correlated with the immune cell composition of blood - I wonder of a blood cell count from the same day is available? Could be in the frame of standard clinical work-up. I am not convinced that we can take this data as a view on the actual immune tumor environment. Differences in data from cancer patients and controls could also be visible in the standard blood cell count.
4. the immune cell composition is also discussed in the benchmarking approach between the LABS and the two commercial kits - as we do not have a ground truth dataset I wonder how the authos can claim better performance of their assay - that needs to be better explained or discussed differently.

Minor points:
1. cfDNA input quality control is only done with qubit quantification. I would recommend to always control for the fragment size profile, e.g. with an Agilent cfDNA tape, to understand the degree of cellular DNA contamination. This is especially relevant if the immune cell composition (mostly blood cells) is reported (line 233ff) which might be heavily biased if the cfDNA input quality is heterogeneous.
2. the CNV on a global level in cfDNA in cancer patients is intensly studied and publishe many times in the last 10 years - the words "unexpectadly" (l. 219) or "surprisingly" (l. 234) sound a bit naive.

**Authors’ response**

**Reviewer #1**:

Comment: In the article "Linear Amplification based Bisulfite Sequencing Enables Ultrasensitive Cancer Detection from Cell-free DNA", the authors describe a new method entitled LABS to detect cancer from cell-free DNA from blood using the detected abnormalities in DNA methylation levels. The paper is well-written, the results are convincing and address an important challenge in the field. There are just few minor points that need to be addressed.

**Response**: We appreciate the reviewer’s positive comments. Please see our point-by-point response to other comments.

Major comments

Comment: 1. The authors make a PCA (fig. 2b) using differentially methylated regions, i.e pre-selecting features that are differential between the two groups. It would be good to see how the PCA looks like if taking the methylation of all TSS from differentially expressed genes. If this does not show the separation in groups it would be good to nuance the statements, confirming the reliability of DNA methylation to correlate with part of the GE changes. Important to note is that all the data can only talk about correlations not causal predictions.

**Response:** We appreciate the reviewer’s suggestions. Indeed, only 41 out of 3188 differentially expressed genes showed differential promoter methylation (percent methylation difference >= 12%, FDR < 0.01). Consistently, the PCA plot by using the TSS methylation levels of all differentially expressed genes showed less clear group separation:

We have changed the statement in our revised manuscript on page 10 to: “*confirming the reliability of promoter DNA methylation based on the LABS data to correlate with at least part of gene expression changes associated with colon cancer*”.

Comment: 2. Related to the remarks "We did not find significant differences in B cell-derived cfDNA among the three groups (Fig. 3d). This finding is consistent with the immune suppressive features of enriched fibroblast cells in the tumor microenvironment of PDAC." It is not clear how the second sentence follows from the first one, this could be explained better, e.g. how does the lack of difference in B-cells relate to immune suppression?

**Response:** We thank the reviewer for pointing this out. The second sentence was mistakenly repeated after Fig. 3d. We have highlighted the original sentence following Fig. 3f in our revised manuscript on page 12.

Comment: 3. It is unclear how suppl. fig 4c should be interpreted. What does "health" mean and how is it computed. And the different fractions using the other methods, do they come from healthy samples? And from which amount of input DNA. It seems also that the link to suppl. fig 4c is missing in the text.

**Response:** We showed in this figure the deconvolution results for 12 samples using 3 different library construction strategies in Supplementary Table 1. The 12 samples were all from the same commercial cfDNA of healthy subjects. We included 2 different input DNA amounts: 1 ng and 0.1 ng (Supplementary Table 1). Since our LABS results on 100 clinical samples showed promising results (Fig. 2 and 3), we then used all 34 samples from healthy controls as the reference in this figure. We have added the citation to Supplementary Fig. 4c in our revised manuscript on page 13, and rewritten the figure legend in the supplementary information on page 8: “*Immune cell type specific deconvolution of cfDNA samples using three different library construction strategies, as shown in Supplementary Table 1. Healthy, deconvolution results from 34 healthy samples as shown in Fig. 2a*.”

Minor comments

4. Figures:

Comment: Legend in 1j is missing. Though the legend is the same as 1i, it would be nice to repeat it, or put it completely to the left, to see that the legend indeed belongs to i and j.

**Response**: Thanks for pointing this out. We have added the legend to Fig. 1j.

Comment: Suppl. Figure 1i seems to miss a legend.

**Response:** Thanks for pointing this out. We have added the legend to Supplementary Fig. 1i.

Comment: "Coverages on both DNA strands were comparable for all three methods (Supplementary Fig. 2c)." I think the authors aim to refer to suppl. fig 1c here.

**Response:** We thank the reviewer for bringing this to our attention. We have corrected this in our revised manuscript on page 8.

Comment: Figure 4c is difficult to follow. Why are there multiple entries per layers, maybe the legend or the figure could be made more explanatory (Suppl. fig 5 for example is more explanatory in this sense)

**Response:** We thank the reviewer for this comment. Each bar in Fig. 4c represents one variable in the random forest model, either a principal component (i.e., TSS methylation or copy number alteration), or a relative percentage of a particular immune cell type. Supplementary Fig. 5a lists the detailed information of the 20 most important variables as shown in Fig. 4c. We have rewritten the legend of Fig. 4c on page 29 of the revised manuscript: “*Variable importance of the random forest model shown in b. Each bar represents one variable, either a principal component or a relative percentage of an immune cell type. Different colors denote different variable types*.”

Comment: It would be informative in suppl figure 2d to label which genes are differentially up- and which are downregulated.

**Response:** As suggested, we have labeled the genes with different colors in Supplementary Fig. 2d, and have added a statement in the figure legend on page 5: “*Genes with upregulated expression in cancer samples are labeled in red, and genes with downregulated expression in cancer samples are labeled in black.*”

Comment: 5. Abbreviations: At page 8 it would be maybe good to introduce again the abbreviations PDAC and CRC for the readers that are less familiar with these terms. Same holds true for COAD and PAAD elsewhere in the result section of the manuscript.

**Response:** As suggested, we have expanded these abbreviations in the revised manuscript on pages 4, 9, and 13.

Comment: 6. I tried to find how CpGs were assigned to genomic context (CDS, intron, UTR etc.) but I think it is not listed in the methods, could it be added?

**Response:** As suggested, we have added this part of methods in the revised manuscript on page 21: “*Assignment of CpGs to different genomic features (e.g., exons, introns, etc.) was done by Homer with default priority list*.”

**Reviewer #2**:

Comment: The manuscript by Cui et at. applied linear amplification, previously developed for single-cell DNA sequencing (e.g. in LIANTI), to DNA methylation sequencing by combining it with bisulfite sequencing. Linear amplification is known to reduce PCR bias and has more even genome coverage. The authors showed the resulting Linear Amplification based Bisulfite Sequencing (LABS) could also reduce the bias in DNA methylation detection compared to traditional PCR-based bisulfite sequencing. The authors then mainly focused on cell-free DNA (cfDNA) application, and showed LABS can be applied to low input (1-2.5 ng) cfDNA to provide multiple features including cancer-specific methylation patterns, copy number alterations, and tissue deconvolution for cancer detection.

Comment: 1. There are numerous cfDNA bisulfite sequencing studies showed the capability to detect CNVs and perform tissue deconvolution. Without more direct head-to-head comparison, it may be premature to say LABS is much better at CNV detection and tissue deconvolution. For example, for those CNVs detected in CRC and PDAC, could normal bisulfite sequencing also detect them? On the other hand, is there any normal cfDNA sequencing done as positive control to show these are indeed CNVs? If the authors break down cancer by stages, do late stage cancers have more CNVs?

**Response:** We thank the reviewer for this comment. Although we did not have direct head-to-head comparison on real clinical cfDNA samples, our results on mESCs and commercial cfDNA samples have demonstrated that the LABS consistently showed larger and more unbiased genomic coverage for low input DNA than the other two commercially available bisulfite sequencing methods. These features of the LABS are critical for determining copy number alterations and tissues of origin. For instance, MethylC-seq and EpiGnome methods both showed >70% duplication levels for 1 ng of input DNA, while the LABS achieved <10% duplication levels (Supplementary Fig. 1g). Our results suggested that MethylC-seq and EpiGnome would lose a much larger part of the genome compared to our LABS protocol, including potential CNVs. On the other hand, we have also showed in Supplementary Fig. 2e that known amplification and deletion markers of CRC from TCGA database could also be detected from our LABS results, further supporting the technical robustness and relevance of this technology in biomarker discovery.

Comment: 2. Fragmentation profile is another important feature of cfDNA. The unbiased LABS should mean it better preserve the fragmentation profile of cfDNA than standard bisulfite. Have the authors looked into that?

**Response:** This is a very good point. Yes, we indeed have investigated the fragmentation profiles. However, bisulfite treatment itself can also fragment cfDNA, thus likely confounding the analysis. For this reason, in the current work we excluded fragmentation profiles for further analysis.

Comment: 3. On the cancer diagnosis part, how is the model performance in different cancer stages? The authors only showed the model for CRC. What about PDAC detection? Since LABS can detect tissue of origin, could it detect CRC and PDAC simultaneously?

**Response:** We would like to clarify that our clinical sample analysis was meant to evaluate the feasibility of applying the LABS to clinical samples. The purpose was therefore to show that we could identify methylation features relevant to CRC in general and potentially cancer-specific features. Future larger clinical studies will be needed to comprehensively investigate 5mC biomarkers for CRC and PDAC using this new technology. For example, at the current sample size, for each stage of CRC, we have 11-15 samples, models would easily be overfitted due to the small sample sizes if we were focused on stage-wise analysis. Similarly, due to the small sample size of PDAC (n = 16), we didn’t show results of PDAC detection model performance. The LABS detected colon-derived cfDNA fragments significantly higher in CRC samples than in non-cancer controls (Fig. 3b), but did not detect significantly higher proportion of pancreas-derived cfDNA fragments (Fig. 3c), possibly caused by small organ size of pancreas and high fibroblast composition of PDAC. The small sample size of the PDAC set could also be a reason. Again, we believe these limitations are not our primary focus of the current work. We were most interested in showing a new technology that could be applied to clinically feasible biospecimens.

Comment: 4. Figure 1j, what is the reason for the peak around 60% GC percentage in LABS? What would standard DNA sequencing look like?

**Response:** Ideally, the genomic coverage should be evenly distributed across the genome, no matter how much the GC percentages are (i.e., no GC content bias). But in reality, strong GC bias can usually be observed, mainly caused by PCR[1]. Here is one example of genomic coverage in standard DNA sequencing from normal white blood cells[1]:

For the LABS, the possible reason is that single-stranded DNA with high or low GC content may have secondary structures or are not stable. However, in Fig. 1j, we would like to emphasize that regions with extreme GC percentages (left and right sides of the curves) also had relatively high sequencing coverages, compared to the other two methods. Therefore, the LABS enables sensitive detection of potential 5mC biomarkers from regions with extreme GC percentages, a significant improvement over these other competing technologies.

Comment: 5. What is the mESCs methylation level detected by EpiGenome and MethylC-seq?

**Response:** We showed the methylation level information in Supplementary Table 1. Here is a figure based on that information:

Comment: 6. Supplementary Fig. 2d, I cannot see a difference between healthy and CRC.

**Response:** The cutoffs we used in this figure are: percent methylation difference ≥ 12%, FDR < 0.01. Since methylation level scale is from 0 to 100%, minor methylation differences can be hard to appreciate. To increase the readability of Supplementary Fig. 2d, we have separated and labeled genes into two different categories: genes that are highly expressed and lowly methylated in cancer samples are marked red, whereas genes that are lowly expressed and highly methylated in cancer samples are marked black.

Comment: 7. Line 270-275, is it talking about Supplementary Fig. 4c, which was never cited in the main text? It's unclear what data is this. Are MethylC-seq and EpiGenome been applied to the same cfDNA cohort as LABS?

**Response:** We thank the reviewer for bringing this to our attention. We have added the figure citation in our revised manuscript on page 13. We showed in this figure the deconvolution results for 12 samples using 3 different library construction strategies in Supplementary Table 1. The 12 samples are all from the same commercial cfDNA of healthy subjects. We have included 2 different input DNA amounts: 1 ng and 0.1 ng (Supplementary Table 1). Since our LABS results on 100 clinical samples showed promising results (Fig. 2 and 3), we used all 34 healthy control samples as our reference in this figure. We have rewritten the figure legend in our revised supplementary information on page 8: “*Immune cell type specific deconvolution of cfDNA samples using three different library construction strategies, as shown in Supplementary Table 1. Healthy, deconvolution results from 34 healthy samples as shown in Fig. 2a*.”

Comment: 8. Some figures are cited wrong. Line 165, Supplementary Fig. 2c should be Supplementary Fig. 1c. Line 290, Supplementary Fig. 5b should be Fig. 4d.

**Response:** Thanks for pointing this out. We have corrected them in our revised manuscript on pages 8 and 14.

**Reviewer #3**:

Comment: Prof Chuan He and colleagues applied their published linear amplification method in another low-input material field, namely cfDNA studies, here also combined with methylation detection. Whereas less-biased amplification is probably advantageous in many settings, I have some concerns about the study design and the strategy that is used to proof this point.

Major concerns:

Comment: 1. Data is presented that show that the assay works on minute amounts of DNA, which is impressive, but not as relevant to the field as discussed here - Normally, 10ng of cfDNA is available from an average blood draw. The far more important question is, if the method is able to detect very early stages or minimal residual disease to be helpful for screening approaches or therapy monitoring. To test sensitivity and specificity of the approach, linear tests with spike-in probes (e.g. with specific genotypes) should be used to proof the advantages from linear amplification over conventional PCR approaches and on cfDNA, and not cell lines (l 119ff, 140ff). The coverage of CpGs is indeed impressive compared to the other two assays, but it needs to be shown in the proposed context.

**Response:** We appreciate the reviewer for this good point. However, we would like to emphasize here that, firstly, cfDNA is a mixture with different sources of DNA fragments, and even 10 ng of cfDNA may only contain < 100 pg ctDNA or DNA fragments from certain cell types; secondly, in real-world clinical settings, often only limited amount of blood is available. Our LABS method can work on as low as 10 pg of input DNA, thus with the capability of enabling detection of underrepresented DNA components (1%) from even 1 ng of cfDNA mixture, making other diagnosis/laboratory tests from the same blood sample possible. In this manuscript, we showcased the results from 1 ng of cfDNA with significant methylation level changes, copy number alterations, and tissue-of-origin differences, providing a robust method for future large clinical studies.

We also did the method comparisons on commercial cfDNA samples with different input DNA amounts, as shown in Supplementary Fig. 1h and 1i. The LABS has much lower duplication levels and higher genomic coverage even from 100 pg of input cfDNA.

Comment: 2. the establishment of biomarkers in a targeted approach would put this study to another level

**Response:** We totally agree with the reviewer. We indeed have such plan as our next step for several cancer types. Nonetheless, in this manuscript, our primate goal is to introduce a new technology that could be applied to clinically feasible biospecimens.

Comment: 3. the immune cell composition in cfDNA is heavily correlated with the immune cell composition of blood - I wonder of a blood cell count from the same day is available? Could be in the frame of standard clinical work-up. I am not convinced that we can take this data as a view on the actual immune tumor environment. Differences in data from cancer patients and controls could also be visible in the standard blood cell count.

**Response:** We thank the reviewer for this comment. Unfortunately, we did not have the blood cell count data for our samples. However, according to a recent research[2], the difference between the ratio of B to T cells expected in the circulation and the ratio of B cell-derived DNA to T cell-derived DNA in the circulation is highly significant, suggesting the necessity of direct deconvolution from cfDNA mixture. Furthermore, information from deconvolution results can be used as independent variables in addition to methylation biomarkers, copy number alterations, and standard blood cell counts.

Comment: 4. the immune cell composition is also discussed in the benchmarking approach between the LABS and the two commercial kits - as we do not have a ground truth dataset I wonder how the authors can claim better performance of their assay - that needs to be better explained or discussed differently.

**Response:** We showed in Supplementary Fig. 4c the deconvolution results for 12 samples using 3 different library construction strategies in Supplementary Table 1. The 12 samples were all from the same commercial cfDNA of healthy subjects. We have included 2 different input DNA amounts: 1 ng and 0.1 ng (Supplementary Table 1). Since our LABS results on 100 clinical samples show promising results (Fig. 2 and 3), we chose to use all 34 samples from healthy controls as our reference in this figure. As we can see from this figure, only the LABS shows high consistency for each cell type component, compared to the other two competing methods.

Minor points:

Comment: 1. cfDNA input quality control is only done with qubit quantification. I would recommend to always control for the fragment size profile, e.g. with an Agilent cfDNA tape, to understand the degree of cellular DNA contamination. This is especially relevant if the immune cell composition (mostly blood cells) is reported (line 233ff) which might be heavily biased if the cfDNA input quality is heterogeneous.

**Response:** We totally agree with the reviewer on this point. The cellular DNA contamination is so important that we indeed did the fragment size profiles for all samples, and only used samples with percentages of small DNA fragments larger than 85%. Here is one example fragment size profile of our excluded samples:

Comment: 2. the CNV on a global level in cfDNA in cancer patients is intensely studied and published many times in the last 10 years - the words "unexpectedly" (l. 219) or "surprisingly" (l. 234) sound a bit naive.

**Response:** Thanks for pointing this out. We have deleted those two words in our revised manuscript on pages 10 and 11.

**Reference:**

1.	Benjamini Y, Speed TP: **Summarizing and correcting the GC content bias in high-throughput sequencing.** *Nucleic Acids Research* 2012, **40:**e72-e72.

2.	Mattox AK, Douville C, Wang Y, Popoli M, Ptak J, Silliman N, Dobbyn L, Schaefer J, Lu S, Pearlman AH, et al: **The Origin of Highly Elevated Cell-Free DNA in Healthy Individuals and Patients with Pancreatic, Colorectal, Lung, or Ovarian Cancer.** *Cancer Discovery* 2023, **13:**2166-2179.

**2^nd^ round**

**Reviewer 1**

In the revised version of the article “Linear Amplification based Bisulfite Sequencing Enables Ultrasensitive Cancer Detection from Cell-free DNA”, the authors describe a new method entitled LABS to detect cancer from cell-free DNA from blood using the detected abnormalities in DNA methylation levels. The authors have addressed all my concerns, although the manuscript would still benefit from further addressing two of my initial major comments. I also added some minor comments that the authors may want to address.

Major comments

1. The authors make a PCA (fig. 2b) using differentially methylated regions, i.e pre-selecting features that are differential between the two groups. The PCA of promoter methylation levels showing all the differentiallly expressed genes is showing quite a clear distinction as well (shown in the eresponse to reviewers). I would suggest the authors to show this figure in the supplement of the manuscript. It is stronger evidence of the association between methylation and GE. First selecting differentially methylated regions and then doing a PCA (as done now) is not meaningful to me as one obviously selects for differences prior to the PCA.

2. It is remains unclear how suppl. fig 4c should be interpreted. I suggest the authors to change the legend and the figure to adress these points below:
- Labeling the groups into one biological group (healthy) and three experimental groups (LABS, methylC and EpiGenome) is confusing. It would be good to change this.
- It is clear now that healthy means the 34 healthy samples. It would be good to add in the legend that these are healthy samples analysed by LABS.
- For each experimental group (LABS, methylC and EpiGenome) it remains unclear if the data represents healthy samples or tumor samples or a mix.

Minor comments

3. Figures:
-Reference to figure 1k seems to be missing in the text.
-Reference to Figure 3f seems to come before reference to 3c-3e
- Figure 4 and the related text: it would be nice to see in the text of the manuscript that the method aims to distinguish CRC from healthy samples, I think this is not really clearly stated.

4. Regarding the sentence: “These identified CNAs from cfDNA were further confirmed as tumor-derived based on previously known CNAs from TCGA database (Supplementary Fig. 2e)”, and the related figure legend. It would be good to add if this TCGA data is coming from the exact same patients analysed by LABS in the current study. If so, a comparison figure between the LABS results and the TCGA results (paired per patient) would be very informative. If the TCGA data is just a global overview of CNVs in CRC and PDAC this should be stated. In that case, it would be nice to see how many of the TCGA related CNVs are seen in at least one patient investigated by LABS.

**Reviewer 2**

The authors have answered my comments

**Reviewer 3**

All concerns have been addressed satisfactorily.

**Authors’ response**

Reviewer #1:

Comment: In the revised version of the article “Linear Amplification based Bisulfite

Sequencing Enables Ultrasensitive Cancer Detection from Cell-free DNA”, the authors

describe a new method entitled LABS to detect cancer from cell-free DNA from blood

using the detected abnormalities in DNA methylation levels. The authors have

addressed all my concerns, although the manuscript would still benefit from further

addressing two of my initial major comments. I also added some minor comments that

the authors may want to address.

Response: We appreciate the reviewer’s positive comments. Please see our point-by-

point response to other comments.

Major comments

Comment: 1. The authors make a PCA (fig. 2b) using differentially methylated regions,

i.e pre-selecting features that are differential between the two groups. The PCA of

promoter methylation levels showing all the differentially expressed genes is showing

quite a clear distinction as well (shown in the response to reviewers). I would suggest

the authors to show this figure in the supplement of the manuscript. It is stronger

evidence of the association between methylation and GE. First selecting differentially

methylated regions and then doing a PCA (as done now) is not meaningful to me as

one obviously selects for differences prior to the PCA.

Response: We appreciate the reviewer’s suggestions. We have put the PCA figure into

our supplementary figure S2e, and cited in the main text accordingly.

Comment: 2. It is remains unclear how suppl. fig 4c should be interpreted. I suggest

the authors to change the legend and the figure to address these points below:

- Labeling the groups into one biological group (healthy) and three experimental groups

(LABS, methylC and EpiGenome) is confusing. It would be good to change this.

- It is clear now that healthy means the 34 healthy samples. It would be good to add in

the legend that these are healthy samples analyzed by LABS.

- For each experimental group (LABS, methylC and EpiGenome) it remains unclear if

the data represents healthy samples or tumor samples or a mix.

Response: We thank the reviewer for these suggestions. We have changed the labels

in Fig. S4c, and added more details in figure legends.

Minor comments

Comment: 3. Figures:

-Reference to figure 1k seems to be missing in the text.

-Reference to Figure 3f seems to come before reference to 3c-3e

- Figure 4 and the related text: it would be nice to see in the text of the manuscript that

the method aims to distinguish CRC from healthy samples, I think this is not really

clearly stated.

Response: We thank the reviewer for bringing these to our attention. We have

corrected the figure reference issues, and added more details of the classifiers in our

revised manuscript.

Comment: 4. Regarding the sentence: “These identified CNAs from cfDNA were further

confirmed as tumor-derived based on previously known CNAs from TCGA database

(Supplementary Fig. 2e)”, and the related figure legend. It would be good to add if this

TCGA data is coming from the exact same patients analyzed by LABS in the current

study. If so, a comparison figure between the LABS results and the TCGA results

(paired per patient) would be very informative. If the TCGA data is just a global

overview of CNVs in CRC and PDAC this should be stated. In that case, it would be

nice to see how many of the TCGA related CNVs are seen in at least one patient

investigated by LABS.

Response: We thank the reviewer for this comment. The patient samples were

collected by ourselves at the University of Chicago, independent of those samples from

TCGA. We used TCGA database as a reference to corroborate our findings. We have

added a statement on page 11 of the revised manuscript: “These identified CNAs from

cfDNA were further confirmed as tumor-derived based on previously known CNAs from

TCGA database, with 10 COAD amplification markers and 9 COAD deletion markers

can be found in at least one CRC patient cfDNA profile”.
